# Spatial Blind Source Estimation of Respiratory Rate and Heart Rate Detection Based on Frequency-Modulated Continuous Wave Radar

**DOI:** 10.3390/s25041198

**Published:** 2025-02-15

**Authors:** Tong Pei, Tao Liao, Xiangkui Wan, Binhui Wang, Danni Hao

**Affiliations:** Hubei Power Grid Intelligent Control and Equipment Engineering Technology Research Center, Hubei University of Technology, Wuhan 430068, China

**Keywords:** FMCW radar, respiratory rate and heart rate, weighted principal component analysis

## Abstract

When detecting respiratory rate and heart rate in an FMCW radar room, there is a lot of static clutter and white Gaussian noise generated by hardware heat loss in the environment, which makes the separation of respiratory and heartbeat signals poor. At the same time, the harmonic component of the respiratory signal in the frequency domain will affect the estimation of heart rate. To solve the above problems, a spatial blind source estimation method was proposed to accurately estimate respiratory heart rate. Firstly, the weighted principal component analysis (WPCA) algorithm was used to extract the features of the target signal from the IF signal, and then the respiratory heart rate signal was reconstructed according to the different features. Then, the multi-signal classification (MUSIC) algorithm is used to convert the respiration and heartbeat signals into the zero domain to avoid the influence of the respective harmonic components on the detection results. The experimental results showed that the accuracy of respiratory rate detection and heart rate detection was 94.51% and 97.79%, respectively. Compared with the traditional algorithm, the proposed method is stable and has higher detection accuracy.

## 1. Introduction

In recent years, non-contact vital sign detection methods have gained attention due to their high precision, strong penetration, and minimal environmental interference. Common non-contact detection methods include infrared detection, radar echo detection, and optical detection, with millimeter wave radar emerging as a primary choice for its advantages.

In the study of human respiratory and heart rate detection using FMCW (Frequency-Modulated Continuous Wave) millimeter wave radar, Liu Luyao et al. employed the Wavelet Packet Decomposition (WPD) algorithm to separate respiratory heart rate signals, effectively mitigating interference from noise [1]. Alizadeh et al. used 77 GHz millimeter wave radar to collect intermediate frequency signals and proposed the MTI-EEMD (Multivariate Empirical Mode Decomposition) algorithm, which outperformed the WPD algorithm, although the separation range remained limited in practical applications [2]. Ding Chuanwei et al. introduced an Ultra-Wideband (UWB) radar system based on variational mode decomposition for respiratory heart rate monitoring, achieving accurate identification of respiratory signals, though with shortcomings in heartbeat signal identification [3]. Tang Liangyong et al. developed the WA-EMD (Wavelet-Assisted Empirical Mode Decomposition) algorithm for the accurate separation of breathing signals but did not address heartbeat signals [4]. Han Che et al. separated heartbeat signals using target signal variance and estimated heart rate with Fast Fourier Transform (FFT) but did not explore respiratory signals in-depth [5].

The development of Ultra-Wideband (UWB) radar technology for medical applications dates back to the 1970s, as discussed in Staderini’s study on UWB radars in medicine [6]. Since then, research has led to minor improvements, yet widespread practical application remains limited. Understanding the reasons behind this slow transition from theoretical advancements to real-world deployment is crucial for assessing the current state of the field.

One of the primary challenges is signal interference and clutter, which has historically hindered accurate vital sign detection in non-contact monitoring applications. While traditional signal processing techniques were developed to mitigate these issues, their effectiveness is often compromised in real-world scenarios due to environmental factors and system noise. Additionally, hardware limitations, such as the high cost of high-resolution radar systems and the need for specialized signal processing hardware, have restricted the adoption of UWB radar-based medical devices in commercial and clinical settings.

Another significant barrier is regulatory approval and standardization. The implementation of UWB radar for medical applications must comply with stringent electromagnetic radiation regulations, which vary across different countries and regions. The lack of universally accepted standards has further delayed the commercialization of these systems.

Moreover, clinical validation and user acceptance remain significant hurdles. Despite advancements in signal processing algorithms, many UWB radar-based health monitoring systems lack large-scale clinical validation studies to demonstrate their reliability and accuracy compared to conventional medical monitoring devices. This lack of validation reduces the willingness of healthcare providers to adopt the technology in critical care environments.

To tackle these challenges, this paper proposes a spatial blind source estimation method that leverages the WPCA algorithm to separate respiratory and heartbeat signals, followed by the MUSIC algorithm for respiratory heart rate estimation, enhancing the accuracy and flexibility of FMCW radar for detecting human vital signs.

## 2. FMCW Radar Respiration and Heart Rate Detection Principle

**Figure 1:**
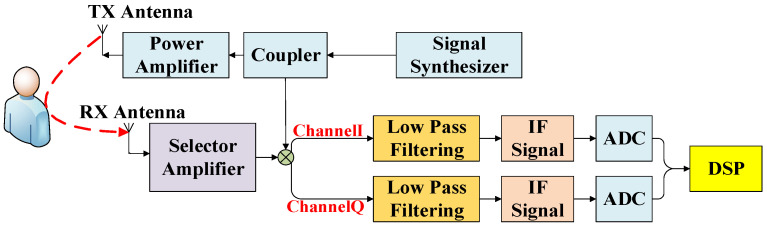
FMCW radar structure diagram.

The FMCW millimeter wave radar system transmits FM continuous waves to detect respiratory and heart rates based on displacement information from the human thoracic cavity, acquiring phase information of the respiratory and heartbeat signals [7,8,9]. The system’s structure, illustrated in Figure 1, starts with synthesizing FMCW pulses in the signal synthesizer. These pulses pass through a coupler to the power amplifier and mixer (shown as a multiplier). The power amplifier sends a transmit signal to the TX antenna, which scatters into space and forms an echo after reflecting off a target, subsequently received by the RX antenna and sent to the frequency selector amplifier. The mixer combines the transmit signal and the receive signal, which are processed in a quadrature (I/Q) dual channel. After low pass filtering, the intermediate frequency (IF) signal is generated, sampled by the ADC, and sent to the DSP module for processing.

**Figure 2:**
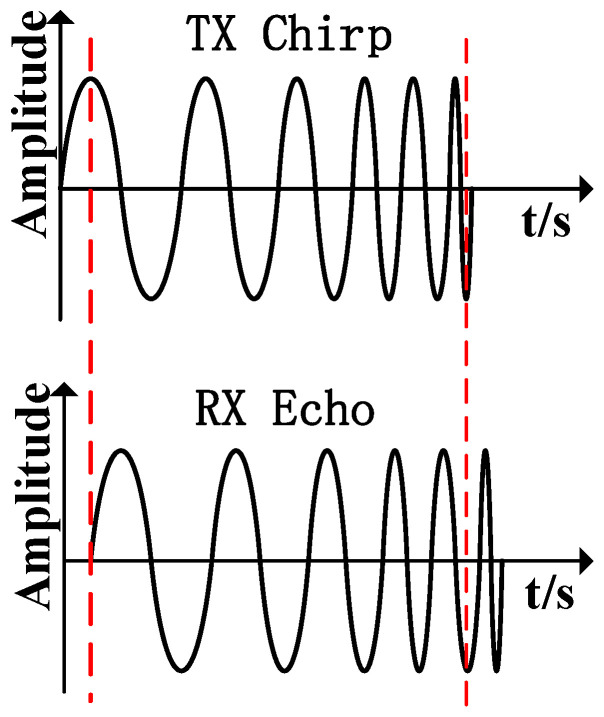
FMCW time domain waveform.

The radar detects targets by transmitting an FMCW with bandwidth B over a period T. The FMCW is a sawtooth wave in the frequency domain, modulated in the time domain by multiple sinusoidal signals with linearly varying frequencies [10]. As depicted in Figure 2, the FMCW signal from the TX antenna can be expressed as follows:

(1) $$S_{T}(t)=A_{T}\cos(2\pi f_{c}t+\pi \kappa t^{2}+\phi (t))$$

where $S_{T}(t)$ is the transmit signal, $A_{T}$ is the transmit power, $f_{c}$ is the linear FM onset frequency, κ is the linear FM slope, and ϕ(t) is the phase noise.

After detecting the target, the waveform of the reflected echo signal during transmission is basically the same as that of the transmitted signal, and the only difference is that the phase information between the echo signal and the transmitted signal produces a change [11,12], as shown in Figure 2. The FMCW signal received by the RX antenna can be expressed as follows:

(2) $$\left\{\begin{array}{l} S_{R}(t)=\alpha A_{T}\cos(2\pi f_{c}(t-t_{d})+\pi \kappa {\left(t-t_{d}\right)}^{2}+\phi (t-t_{d}))\\ t_{d}=2R(t)/c \end{array}\right.$$

where $S_{R}(t)$ is the received signal, α is a constant factor, $t_{d}$ is the time delay between the transmitted and received signals, R(t) is the radial distance between the radar and the human body, and c is the speed of light.

**Figure 3:**
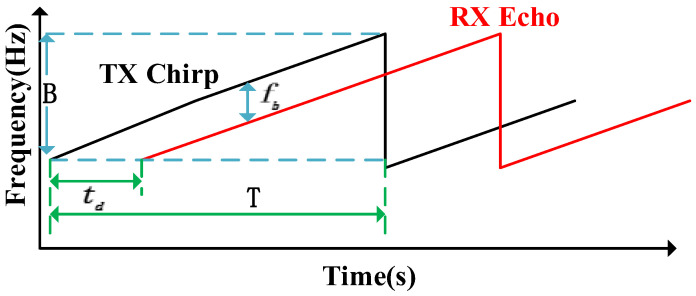
FMCW time–frequency domain diagram.

The human chest cavity experiences displacement changes from breathing and heartbeats, resulting in a time delay in electromagnetic wave propagation and phase shifts in the echo signal. This phase difference between transmitted and received signals is mixed in a frequency mixer to create an IF signal that reflects chest motion [13]. The IF signal, illustrated in Figure 3, indicates the frequency difference between multiple transmit and receive signals. Consequently, the IF signal can be represented as follows:

(3) $$Y(t)=A_{R}\exp(j(2\pi f_{b}t+\phi_{b}(t)+\Delta \phi (t)))$$

where Y(t) is the IF signal, $A_{R}$ is the received signal power, $f_{b}$ is the frequency of the IF signal, $\phi_{b}(t)$ is the phase of the IF signal, and Δϕ(t) is the residual phase noise.

The displacement of the chest cavity due to respiratory motion in normal adults is around 4–12 mm, and the displacement caused by heartbeat is around 0.2–0.5 mm, and the residual signal noise is negligible at this tiny displacement distance [14,15,16]. Therefore, it can be obtained that the small distance change in the subject and the mid-frequency phase signal change can be expressed as follows:

(4) $$\Delta \phi_{b}=\frac{4\pi }{\lambda }\Delta R$$

where $\Delta \phi_{b}$ is the mid-frequency phase signal change, ΔR is the minor distance change, and λ is the wavelength.

During a single FMCW cycle, the radar transmits multiple sets of linear pulses (Chirp), and each Chirp acquires a set of distance versus phase variations [17,18]. All the information acquired by multiple sets of Chirp constitutes the detected signal during that FMCW cycle, which is algorithmically processed to obtain the desired target signals, i.e., respiratory and heartbeat signals [19,20].

## 3. Respiration and Heartbeat Signal Processing

### 3.1. Overall Structure of Algorithm

**Figure 4:**
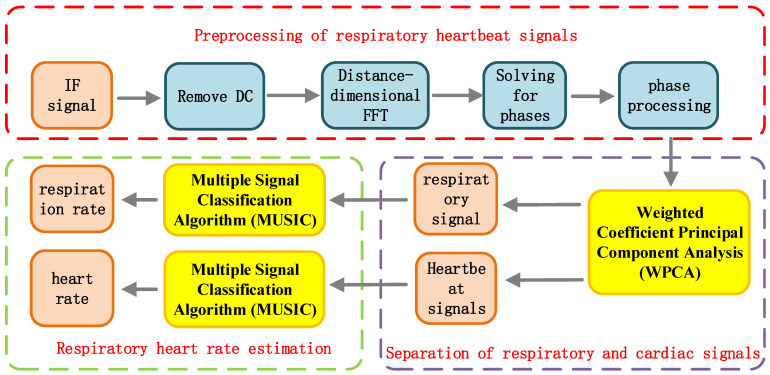
Overall structure of MUSIC algorithm.

Figure 4 illustrates the processing flow for respiratory heart rate detection using FMCW radar, which comprises three parts: respiration and heartbeat signal pre-processing, signal separation, and heart rate estimation. The pre-processing stage involves operations on the IF signal obtained from the FMCW radar, including DC removal, distance dimension FFT, phase solving, and phase processing. The signal separation stage utilizes the WPCA algorithm to separate the respiratory and heartbeat signals from the phase-processed signal. Finally, the heart rate estimation employs the MUSIC algorithm to estimate the respiratory rate and heart rate from the separated signals, respectively.

### 3.2. Pre-Processing of Respiration and Heartbeat Signals

Since the FMCW radar continuously consumes power during the detection process, the FMCW radar hardware board heats up and generates a DC component. At the same time, the DC component leads to thermal power loss, which in turn generates Gaussian white noise and affects the detection of the target signal. Therefore, the DC component removal step is first taken for the IF signal as shown in the following equation:

(5) $$g^{\prime }(t)=g(t)-\bar{g(t)}$$

where $\bar{g(t)}$ is the original IF signal, $\bar{g(t)}$ is the mean value of the IF signal, and $g^{\prime }(t)$ is the IF signal after removing the DC component.

The signal after removing the DC component is then outputted by the distance–dimensional FFT with the corresponding real and imaginary parts, and the thoracic phase signal is solved by the inverse tangent function, as shown in Equation (6).

(6) $$l\phi_{b}(t)=\arctan(Q(t)/I(t))$$

In Equation (6), I(t) is the real data, Q(t) is the imaginary data, and $l\phi_{b}(t)$ is the thoracic phase signal.

The tangent function is monotonically increasing in (−π/2,π/2), while in (0,π), there is a sudden change from positive to negative when the phase value is near π/2. Therefore, the phase obtained by the inverse tangent method needs to be unwound, as shown in Equation (7) below.

(7) $$\varphi (t)=\left\{\begin{array}{ll} l\phi_{b}(t)-2\pi & ,l\phi_{b}(t)-l\phi_{b}(t-1)>\pi \\ l\phi_{b}(t) & ,\left|l\phi_{b}(t)-l\phi_{b}(t-1)\right|\leq \pi \\ l\phi_{b}(t)+2\pi & ,l\phi_{b}(t)-l\phi_{b}(t-1)<-\pi \end{array}\right.$$

In Equation (7), φ(t) is the chest phase signal after unwinding.

Compared with the ambient clutter signal, the signal-to-noise ratio of the thoracic phase signal is very small. Therefore, differential processing of the de-wrapped thoracic phase signal is required to enhance the signal-to-noise ratio of the thoracic phase signal, as shown in Equation (8).

(8) $$\varphi^{\prime }(t)=\varphi (t)-\varphi (t-1)$$

### 3.3. Separation of Respiratory and Cardiac Signals

In the proposed method, the eigenvalue threshold selection in WPCA and the frequency estimation approach in MUSIC are crucial for achieving accurate respiratory and heart rate detection. The threshold for eigenvalue selection in WPCA was determined based on empirical analysis and signal characteristics, ensuring the effective separation of respiratory and cardiac signals.

The eigenvalues in WPCA represent the variance of principal components in the signal. In this study, the threshold for selecting the significant eigenvalues was determined based on the following considerations:

Signal Energy Distribution: The eigenvalues corresponding to respiratory and heartbeat signals exhibit a significantly larger magnitude compared to static clutter and Gaussian white noise. By analyzing the spectral energy distribution of the chest phase signal, we identified that eigenvalues within the range of 1.88 to 2.44 correspond to respiratory signals, whereas those within 1.06 to 1.59 correspond to heartbeat signals. This range ensures that the extracted components primarily contain physiological information rather than noise.

Empirical Validation: Through multiple experimental trials, we observed that selecting eigenvalues below the lower bound resulted in excessive noise interference, while selecting values above the upper bound led to the loss of critical signal components. The final threshold range was established by optimizing the trade-off between noise suppression and signal retention.

Physiological Constraints: The respiratory and heart rate frequencies typically fall within 0.1–0.5 Hz and 0.8–2 Hz, respectively. The selected eigenvalue range ensures that the separated signals remain within these physiological constraints, thereby enhancing the accuracy of subsequent frequency estimation.

The MUSIC algorithm was employed to enhance the accuracy of respiratory and heart rate estimation by transforming the reconstructed signals into the spatial domain. The selection of the number of angle points and covariance matrix estimation method was guided by the following:

Resolution Considerations: The number of angle points was set to 1024, which provides sufficient resolution to detect subtle variations in respiratory and cardiac signals while minimizing computational complexity.

Noise Subspace Determination: The eigenvector space decomposition was performed based on the covariance matrix of the signal. The noise subspace dimension was determined using the Akaike Information Criterion (AIC) and Minimum Description Length (MDL) methods, ensuring that the dominant signal components were preserved while suppressing irrelevant noise.

Experimental Validation: The MUSIC spectrum was analyzed across multiple test scenarios, and the selected parameters consistently yielded peak frequency estimations that closely matched the ground truth provided by the reference monitoring device.

By integrating these parameter selection strategies, the proposed method effectively balances signal separation accuracy and computational efficiency, making it suitable for practical applications in non-contact vital sign monitoring.

**Figure 5:**
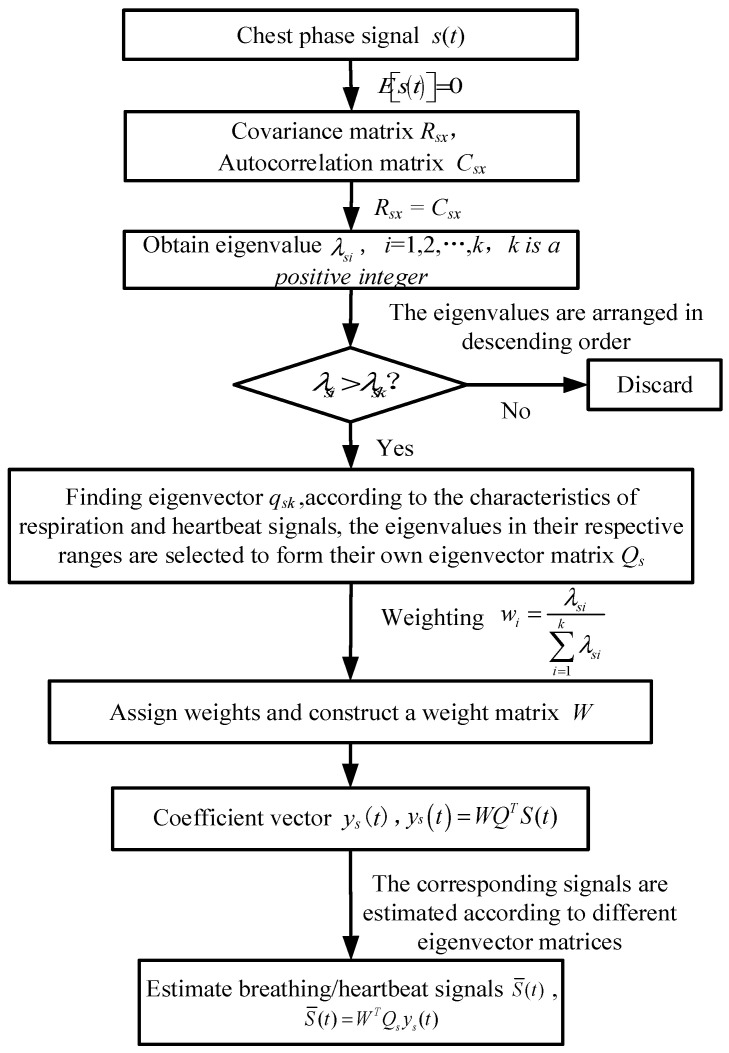
Flowchart of WPCA separating respiration and heartbeat signals.

In addition to the target signal [21,22], the chest phase signal with enhanced SNR also contains static clutter signal and Gaussian white noise and other interference signals in the detection environment. The WPCA algorithm is used to separate the respiratory signal and heartbeat signal from the above interference signals, and the separation process is shown in Figure 5.

The WPCA algorithm is an improvement of the principal component analysis (PCA) algorithm, based on which the weight coefficient matrix is introduced, and the large eigenvalues are given different weights according to the numerical differences, so that the approximate signal can approximate the real signal more accurately. The advantage is that the factor that the eigenvectors corresponding to different eigenvalues have different weights in the original signal is fully considered, and the difference in the selected eigenvalues is reflected in the approximation signal. The implementation principle is as follows:

Using the thoracic phase signal as the input signal s(t), the covariance and autocorrelation matrices are as follows:

(9) $$\left\{\begin{array}{l} R_{sx}=E[(s(t)-\mu_{sx}){\left(x(t)-\mu_{sx}\right)}^{H}]\\ C_{sx}=E[s(t)s^{H}(t)] \end{array}\right.$$

where $R_{sx}$ is the covariance matrix of the signal vector s(t), $C_{sx}$ is the autocorrelation matrix of the signal vector s(t), and $\mu_{sx}$ is the mean of the signal vector s(t).

The covariance matrix and autocorrelation matrix of the signal vector s(t) are equal and are denoted as follows:

(10) $$R_{sx}=C_{sx}$$

Let $\lambda_{sk}$ be the kth eigenvalue of $R_{sx}$, $q_{sk}$ be the kth eigenvector of $R_{sx}$, and the eigenvector $q_{sk}$ is also normalized. Therefore, M eigenvectors can be constructed into eigenvector matrix (6), whose specific expression is as follows:

(11) $$\left\{\begin{array}{l} Q_{s}=[q_{s1},q_{s2},\cdots ,q_{sM}]\\ Q_{s}^{T}Q_{s}=I \end{array}\right.$$

A specific expression for the thoracic phase signal s(t) can be obtained as follows:

(12) $$\begin{array}{ll} s(t) & =[q_{s1},q_{s2},\cdots ,q_{sM}]y_{s}(t)\\ & =Q_{s}y_{s}(t) \end{array}$$

where $y_{s}(t)$ is the coefficient vector of the thoracic phase signal.

The eigenvalues of the thoracic phase signal can be obtained after the KL transform with the following expression:

(13) $$\begin{array}{ll} E[y_{s}(t)y_{s}^{T}(t)] & =diag(\lambda_{s1},\lambda_{s2},\cdots ,\lambda_{sM})\\ & =\Lambda_{s} \end{array}$$

Each characteristic value of the chest phase signal can be obtained from Formula (16). Since the signal corresponding to the large eigenvalues is the desired respiration and heartbeat signal, the eigenvalues are arranged in ascending order by the serial number, and again only the first K coefficients of the coefficient vector are retained, i.e., the first K large eigenvalues are retained, where K < M. By retaining different large eigenvalues, the eigenvector matrices $Q_{sR}$ for respiratory signals and $Q_{sH}$ for heartbeat signals can be constructed. Unlike the PCA algorithm, WPCA assigns weights to the eigenvector matrices $Q_{s}$ according to the retained large eigenvalues. The expression for weight calculation is given in the following equation:

(14) $$w_{i}=\frac{\lambda_{si}}{\sum_{i=1}^{K}\lambda_{si}}$$

where $w_{i}$ is the weight of the eigenvector corresponding to the *i*-th eigenvalue.

Therefore, the respiratory signal $s_{R}(t)$ and the heartbeat signal $s_{H}(t)$ can be approximated as $\bar{s_{R}}(t)$ and $\bar{s_{H}}(t)$, which are expressed as follows:

(15) $$\left\{\begin{array}{l} \bar{s_{R}}(t)=\sum_{i=1}^{K}w_{iR}y_{si}(t)Q_{sR}\\ \bar{s_{H}}(t)=\sum_{i=1}^{K}w_{iH}y_{si}(t)Q_{sH} \end{array}\right.$$

where $w_{iR}$ is the weight of the eigenvector corresponding to the *i*-th eigenvalue of the respiratory signal, and $w_{iH}$ is the weight of the eigenvector corresponding to the *i*-th eigenvalue of the heartbeat signal.

According to the values of different eigenvalues, the corresponding eigenvectors are weighted to make the features more prominent, so as to reconstruct the signal more accurately. In addition, the reference of the weight coefficient improves the sensitivity of the PCA algorithm to the detailed information between different features in the signal. Finally, the selection conditions for the eigenvalues of the constructed eigenvector matrix $Q_{sR}$ of the respiratory signal and the eigenvector matrix $Q_{sH}$ of the heartbeat signal should meet: the frequency of the reconstructed respiratory signal is within the range of 0.1 Hz~0.5 Hz, and the frequency of the heartbeat signal is within the range of 0.8 Hz~2 Hz.

### 3.4. Respiratory Heart Rate Estimation

**Figure 6:**
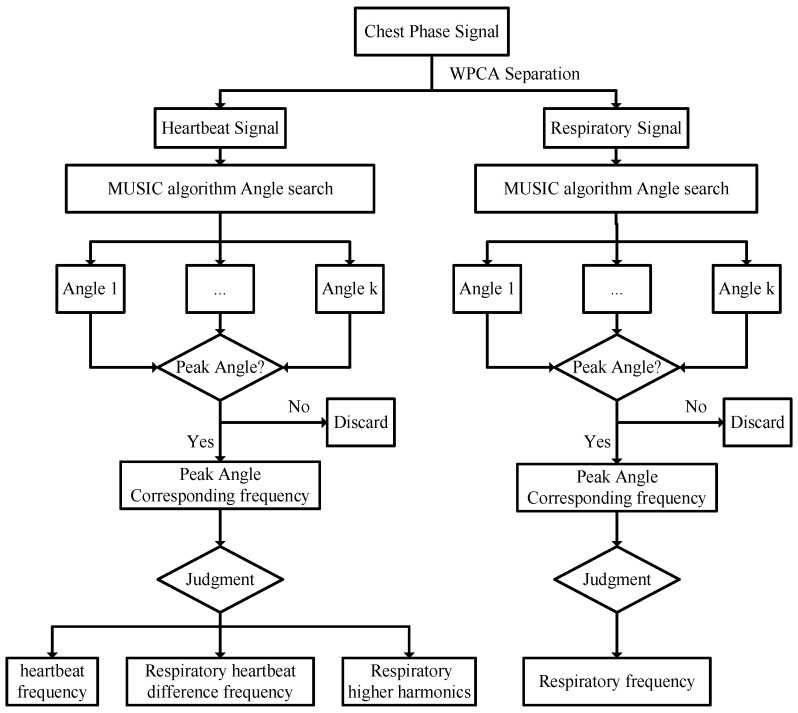
MUSIC algorithm estimation flow chart of respiratory heart rate.

After WPCA separation and reconstruction of respiratory and heartbeat signals, the harmonic components of respiratory and heartbeat signals before separation affect frequency estimation, respectively. The MUSIC algorithm, as a spatial spectrum estimation algorithm, converts time domain signals to space domain. The corresponding frequency is derived from the maximum convergence angle of the energy, and the specific flow rate is shown in Figure 6.

The MUSIC algorithm takes the respiratory signal or heartbeat signal separated by the WPCA algorithm as the input signal ls(t), and its algorithm is implemented in the following way:

Firstly, the input signal ls(t) is intercepted into a signal m(t) of length N∗M, which is the data matrix constructed into N∗M. At this point, signal m(t) can be expressed as follows:

(16) $$\begin{array}{ll} m(t) & =\sum_{i=1}^{M}ls_{i}(t)a(\omega_{i})+v(t)\\ & =Als(t)+n(t) \end{array}$$

where $a(\omega_{i})$ is the direction vector, v(t) is the noise vector, A is the angle vector, and n(t) is the total noise vector. The direction vector $a(\omega_{i})$ is represented as follows:

(17) $$a(\omega_{i})={\left[1,e^{-j2\pi \omega_{i}},\ldots ,e^{-j2\pi (N-1)\omega_{i}}\right]}^{T}$$

where $\omega_{i}$ is the corresponding angle. Therefore, the angle vector A can be expressed as follows:

(18) $$A=[a(\omega_{1}),\ldots ,a(\omega_{N})]$$

After the data matrix is constructed, the eigenvector space U and the noise subvector $U_{N}$ are solved. Then, divide [0,2π] into angle points with number $N_{w}$ and construct the cost function $P_{w}$ as follows:

(19) $$P_{w}=\frac{1}{A^{H}(w)U_{N}U_{N}^{H}A(w)}$$

where $A^{H}(w)$ is the covariance matrix of the search angle vector A(w), and $U_{N}^{H}$ is the covariance matrix of the noise subvector $U_{N}$.

As a result, the frequency corresponding to the peak angle of the cost function *P_w_* is found as the frequency of the target signal under the estimation of the MUSIC algorithm. The estimated respiratory signal frequency $f_{br}$ and heartbeat signal frequency $f_{hr}$ are used to obtain respiratory rate (RR) and heart rate (HR), which are given by the following equation:

(20) $$\left\{\begin{array}{l} RR=f_{br}\times 60\;\min^{-1}\\ HR=f_{hr}\times 60\;\min^{-1} \end{array}\right.$$

## 4. Experimental Results and Analysis

**Figure 7:**
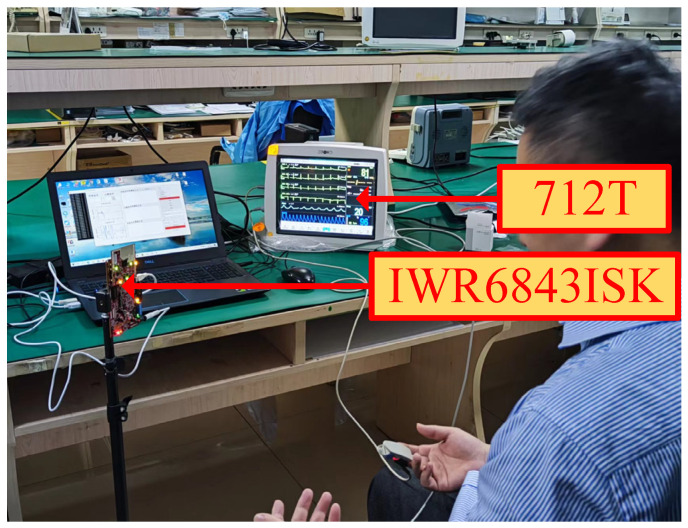
Scenario diagram of respiratory heart rate detection.

This experiment employed the millimeter wave radar device IWR6843ISK manufactured by TI (with an operating frequency band of 60–64 GHz and adopting the FMCW modulation mode), which possesses a radar array element featuring 3 transmitters and 4 receivers. The experimental configuration was set as a single-transmit and single-receive mode (TX1-RX1), and the ADC sampling points were set to 128. The experiment was carried out in an electromagnetic shielding chamber with a temperature of (23 ± 1) °C and a relative humidity of (50 ± 5)%. The specific experimental scenario is depicted in Figure 7. The subjects sat motionless at a position 1 m in front of the IWR6843ISK millimeter wave radar sensor, were required to maintain natural breathing and have their trunks facing the central axis of the radar array, and wore the 712T patient monitor produced by Wuhan Sichuang Electronics Co., Ltd. as a reference device. Its detection values were regarded as the reference values for this experiment, that is, the true values. The following are the experimental hypotheses: (1) The undulation of the human thoracic cavity is the main source of respiratory/heartbeat signals. (2) The environmental multipath interference can be neglected. (3) The time synchronization error between the monitor and the radar signal is ≤10 ms.

### 4.1. Respiration and Heartbeat Signal Pre-Processing Results

**Figure 8:**
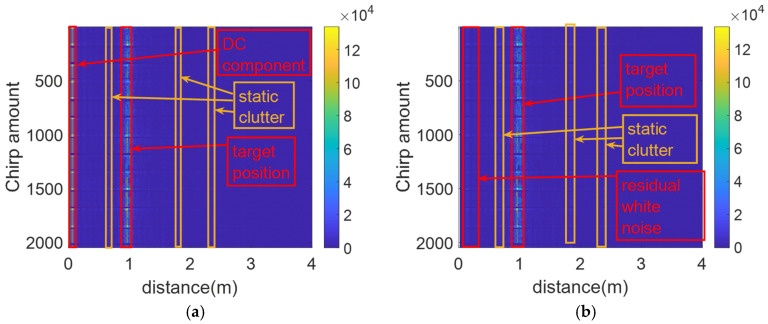
Diagram of DC component processing results. (**a**) Plot of results before removal of DC component; (**b**) plot of results after removal of DC component.

During the detection process, the FMCW radar continuously consumes power, resulting in the heating of the radar hardware board, which causes the phenomenon of DC drift and generates Gaussian white noise. Therefore, in the pre-processing step, the main focus is on the removal of the direct current (DC) component to reduce the impact of DC drift on the detection results. The processing results are shown in Figure 8.

In Figure 8a, the DC component and static clutter are clearly present. While in Figure 8b, the DC component was removed after the DC removal step. Despite the removal of the DC component in the pre-processing step, there are still interfering signals such as Gaussian white noise and static clutter, which affect the subsequent separation of the respiration and heartbeat signal.

### 4.2. Respiration and Heartbeat Signal Separation Results

After pre-processing, the DC component of the interference signal was removed, but there are still static clutter and residual white noise interference signals. The WPCA algorithm is used to separate the respiration and heartbeat signal from the pre-processed chest phase signal.

**Figure 9:**
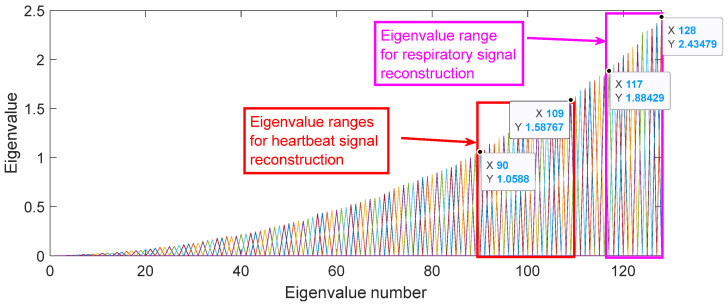
Plot of eigenvalue ranges selected for WPCA-reconstructed target signals.

Due to the presence of respiratory signal, heartbeat signal, static clutter signal, and Gaussian white noise in the chest phase signal, their specific performance in the WPCA processing results is the difference in the size of the eigenvalues. Compared with static clutter and Gaussian white noise, the target signal is obviously obtained by the WPCA algorithm, so the target signal should have a larger eigenvalue. Therefore, the range of characteristic values of reconstructed respiratory signals and heartbeat signals can be obtained, as shown in Figure 9.

**Figure 10:**
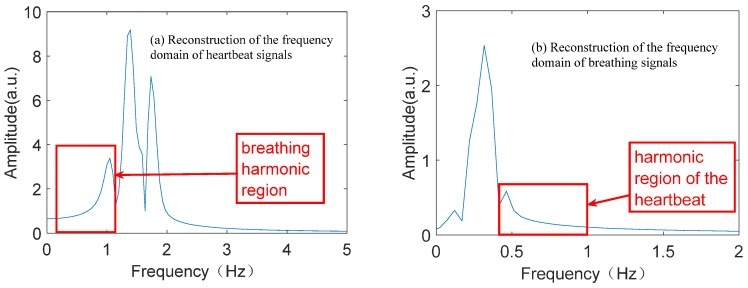
WPCA reconstruction of respiration and heartbeat signal result map.

Figure 9 and extensive experiments on respiration and heartbeat signal reconstruction show that the eigenvalues for reconstructed heartbeat signals range from 1.06 to 1.59, while those for reconstructed respiratory signals range from 1.88 to 2.44. The frequency domain results for these reconstructed signals, based on the selected eigenvalue range, are presented in Figure 10.

In Figure 10a, the respiratory harmonics are present on the left side of the main peak of the heartbeat. And in Figure 10b, the heartbeat harmonics exist on the right side of the main peak of breathing. As can be seen from Figure 10, it is difficult to eliminate the influence of the respective harmonics on each other’s frequency estimation by merely separating and reconstructing the respiration and heartbeat signals through the WPCA algorithm. The effect is specifically manifested in the fact that the presence of harmonics will shift the frequency corresponding to the main peak value, making the acquired main peak frequency have some error with the real main peak frequency.

### 4.3. Respiratory Heart Rate Estimation Results

**Figure 11:**
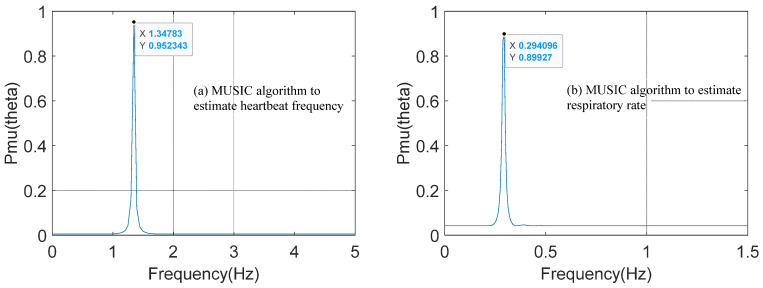
MUSIC algorithm to estimate respiratory heart rate.

Although the effects of static clutter and Gaussian white noise are eliminated, the effects of harmonic components of the respective signals cannot be avoided. Therefore, the MUSIC algorithm is used to estimate the frequency of respiratory and heartbeat signals. The estimated results are shown in Figure 11.

As shown in Figure 11, after the respiration and heartbeat signal is converted to the spatial domain by the MUSIC algorithm, the biased peaks caused by respiratory and heartbeat harmonics have disappeared. In Figure 11a, only a prominent peak for the heartbeat signal is present, and the angle of this peak corresponds to a heartbeat frequency of 1.35 Hz, as estimated by the MUSIC algorithm. Similarly, in Figure 11b, a single prominent peak for the respiratory signal is visible, with the peak angle corresponding to a respiratory frequency of 0.29 Hz. Using Equation (20), the respiration rate is calculated as 17 BPM, and the heart rate as 81 BPM.

### 4.4. Analysis of Experimental Test Results

**Figure 12:**
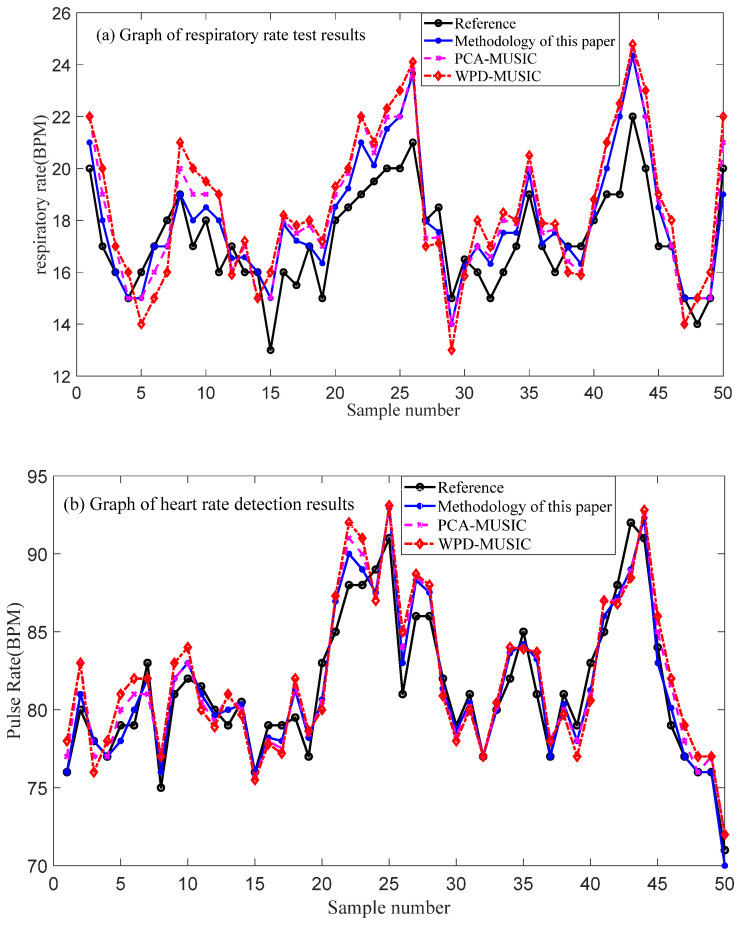
Analysis of detection results.

The sample size of this experiment is 50, which consists of 25 healthy adult male experimental data and 25 healthy adult female experimental data, all of which are in the age range of 20 to 25 years old. In addition, the PCA algorithm and the WPD algorithm among the traditional algorithms are selected as the comparison algorithms of this paper’s method, and the results of their respiratory heart rate detection are shown in Figure 12.

In Figure 12, compared with the reference value of 712T commercial device, the detection result of this paper’s method is closer to the reference value, followed by the detection value of the PCA-MUSIC method, and the detection result of the WPD-MUSIC method deviates the most. Therefore, it can be preliminarily determined that the method of this paper has better accuracy of respiratory rate and heart rate detection. In order to quantitatively reflect the accuracy value of each method, the accuracy calculation for the detection results was taken as in Equation (21).

(21) $$E_{r}=\frac{\left|M_{v}-R_{v}\right|}{R_{v}}\times 100\%$$

where $E_{r}$ is the detection error, $M_{v}$ is the total detection value of all experiments, and $R_{v}$ is the total reference value of all experiments. The comparative analysis of the detection results is shown in Table 1 below.

As can be seen from Table 1, when the same estimation algorithm is used, the method proposed in this paper has the highest accuracy in detecting respiratory rate and heart rate. The experimental results show that compared with the traditional algorithm, the variance of the detection results of this method is minimal (breathing rate is 1.05, heart rate is 1.52). It can also effectively suppress static clutter and white noise and other interference signals, which is suitable for the monitoring of the vital signs of burn patients and drivers.

## 5. Discussion

Finally, the performance of the proposed method in this paper is verified through experiments, and the advantages of this paper’s method compared with traditional algorithms are as follows:

(i) The method is able to correlate the feature values so as to isolate the respiration and heartbeat signal more accurately.
(ii) The method further attenuates the interference signals such as static clutter and residual white noise through different feature weights.
(iii) The method in this paper eliminates the influence of the respective harmonics on the frequency estimation by transforming the reconstructed respiration and heartbeat signal into the null domain.

And from the experimental results in Table 1, it can be seen that the accuracy of FMCW radar respiratory heart rate detection by the method of this paper is better than the traditional algorithm.

While WPCA-MUSIC outperforms PCA-MUSIC and WPD-MUSIC, it relies on eigenvalue-based feature extraction, which assumes a clear distinction between signal and noise. In dynamic environments, this assumption may not always hold, affecting robustness. Machine learning-based methods, such as CNNs and RNNs, have shown promise in FMCW radar-based vital sign detection by learning complex signal patterns. However, these approaches require large, labeled datasets and high computational power, making them less suitable for real-time applications. Future work could explore hybrid approaches, integrating WPCA with deep learning-based feature extraction to enhance robustness, and benchmark WPCA-MUSIC against state-of-the-art deep learning methods for further optimization.

WPCA-MUSIC involves matrix decomposition, which may introduce computational overhead. However, given that vital sign signals reside in a low-dimensional space, the computational burden remains manageable. Future optimizations include the following:

Parallel Computing: Utilizing GPU acceleration or multi-threading to enhance execution speed.

Algorithmic Simplification: Applying randomized singular value decomposition (RSVD) to reduce computation time.

Embedded System Deployment: Optimizing for low-power FPGA or ARM-based edge devices for real-time feasibility.

Future work will focus on benchmarking the execution time of WPCA-MUSIC on different hardware platforms and exploring real-time optimizations. A trade-off analysis between computational complexity and detection accuracy will also be conducted to determine the best balance for practical deployment.

Experiments were conducted in controlled environments, but real-world conditions introduce ambient noise, multipath effects, and subject motion, which may impact accuracy. Key challenges include the following:

Environmental Noise: Interference from wireless devices and power sources.

Multipath Effects: Phase distortions due to reflections from surrounding objects.

Subject Motion: Small movements affecting signal stability.

Future work will focus on adaptive filtering, MIMO radar integration, and real-world testing in hospital wards, moving vehicles, and outdoor environments to improve robustness.

These enhancements will ensure that WPCA-MUSIC is better suited for practical deployment in diverse application scenarios.

## 6. Conclusions

In this paper, a spatial blind source estimation method is proposed to solve the problem that interference signals affect the estimation of respiratory heart rate in FMCW millimeter wave radar. Firstly, the respiratory heart rate signal is separated and reconstructed from the thoracic phase signal by the WPCA algorithm, and then the target signal is converted by the MUSIC algorithm to estimate the respective frequency in the air domain. The experimental results show that the accuracy of this method is better than the traditional method, and it has a better inhibition effect on the interference signal. The method presented in this paper can accurately detect respiratory heart rate in multi-interference environment, which is conducive to the practical application of a FMCW millimeter wave radar respiratory heart rate detection system in multiple scenarios.

## Figures and Tables

**Table 1:** Comparative analysis of testing results.

Parametric	IWR6843ISK					
	WPD-MUSIC		PCA-MUSIC		WPCA-MUSIC	
	RR	HR	RR	HR	RR	HR
standard deviation	1.61	1.96	1.28	1.60	1.02	1.23
variance	2.58	3.84	1.63	2.57	1.05	1.52
accuracy	89.48%	95.72%	92.05%	96.22%	94.51%	97.79%

## Data Availability

The original contributions presented in this study are included in the article. Further inquiries can be directed to the corresponding author.
